# Association between *INADL* genetic variant and a subgroup with high risk for TMD in the OPPERA study

**DOI:** 10.1186/1744-8069-10-S1-P5

**Published:** 2014-12-15

**Authors:** Shad B Smith, Eric Bair, Wei Xue, Gary D Slade, Ronald Dubner, Roger B Fillingim, Joel D Greenspan, Richard Ohrbach, Charlie Knott, Luda Diatchenko, William Maixner

**Affiliations:** 1 Center for Pain Research and Innovation, University of North Carolina, Chapel Hill, NC 27599, USA; 2 Department of Biostatistics, University of North Carolina at Chapel Hill, Chapel Hill, NC 27599, USA; 3 Department of Dental Ecology, University of North Carolina at Chapel Hill, Chapel Hill, NC 27599, USA; 4 Department of Epidemiology, University of North Carolina at Chapel Hill, Chapel Hill, NC 27599, USA; 5 Department of Neural and Pain Sciences, and Brotman Facial Pain Center, University of Maryland Dental School, Baltimore, MD 21201, USA; 6 Department of Community Dentistry and Behavioral Science, University of Florida, Gainesville, FL 32610, USA; 7 Department of Oral Diagnostic Services, University at Buffalo, Buffalo, NY 14214, USA; 8 Battelle Memorial Institute, Durham, NC 27713, USA; 9 Alan Edwards Centre for Research on Pain, McGill University, Montreal, Quebec H3A 1G1, Canada; 10 Department of Pharmacology, University of North Carolina at Chapel Hill, Chapel Hill, NC 27599, USA

## Background

A major impediment to addressing the epidemic of persistent pain conditions is the development of classification procedures that capture the mosaic of signs and symptoms that accompany these disorders. According to the bio-psycho-social model, the measurable features of an idiopathic pain condition such as temporomandibular disorder (TMD) are associated with abnormalities in sensory, psychological, neuroimmune, and autonomic systems, which arise due to the interaction of genetic and environmental risk factors. Using a high-dimensional dataset derived from a large TMD case-control study, we have developed a new method to integrate clinically assessed intermediate phenotypes across bio-psycho-social domains, clustering subjects in a manner that provides clinically useful prognostic and diagnostic information. We performed a candidate gene association study to identify genes that influence cluster assignment in order to characterize the genetic determination of these clusters.

## Materials and methods

Cluster analysis was performed to identify subgroups within the Orofacial Pain: Prospective Evaluation and Risk Assessment (OPPERA) study, which included 1,031 TMD cases and 3,247 controls. TMD status was confirmed using the Research Diagnostic Criteria for TMD (RDC/TMD); each participant was also assessed for psychological characteristics, medical history, and sensitivity to experimental pain. Supervised 3-means clustering was applied to the pain sensitivity and psychological data using the 15 features most strongly associated with TMD. Subjects were genotyped using a candidate gene panel of 2,924 single nucleotide polymorphisms (SNPs) corresponding to 358 pain-relevant genes. We assessed association between cluster identity and SNP genotypes using logistic regression.

## Results

In the contrast between Cluster 1 (n=1180, characterized by low pain sensitivity and low psychological distress) and Cluster 3 (n=273, high pain, high distress), the strongest association was with the SNP rs2498982 (minor allele frequency = 0.39), in the *INADL* gene (standardized OR=1.57, p=1.1x10^-5^). This gene is a scaffolding protein regulating tight junctions in sensory neurons, including interactions between channels involved in nociception such as ASIC3. No statistically significant (after Bonferroni correction) associations were observed in the contrast of Cluster 2 (n=1571, moderate pain, low distress) with Cluster 3 although the strongest signal (p=4.4x10^-4^) was again observed in *INADL*, indicating this gene may distinguish individuals in Cluster 3, with the highest risk of TMD. We also identified other genes potentially contributing to molecular pathways affecting cluster assignment.

## Conclusions

Using a novel clustering method to classify individuals into diagnostic categories, we have identified a genetic variant in *INADL* associated with the subgroup with the highest risk for TMD.

## Disclosures

Smith, Fillingim, Slade, Diatchenko, and Maixner declare financial relationships with Algynomics, Inc.

